# Characterization of the Opp Peptide Transporter of *Corynebacterium pseudotuberculosis* and Its Role in Virulence and Pathogenicity

**DOI:** 10.1155/2014/489782

**Published:** 2014-05-08

**Authors:** Pablo M. R. O. Moraes, Nubia Seyffert, Wanderson M. Silva, Thiago L. P. Castro, Renata F. Silva, Danielle D. Lima, Raphael Hirata, Artur Silva, Anderson Miyoshi, Vasco Azevedo

**Affiliations:** ^1^Instituto de Ciências Biológicas, Universidade Federal de Minas Gerais, 31270-901 Belo Horizonte, MG, Brazil; ^2^Instituto de Ciências da Saúde, Universidade Federal da Bahia, 40210-340 Salvador, BA, Brazil; ^3^Faculdade de Ciências Médicas, Universidade Estadual do Rio de Janeiro, 21941-901 Rio de Janeiro, RJ, Brazil; ^4^Instituto de Ciências Biológicas, Universidade Federal do Pará, 66075-110 Belém, PA, Brazil

## Abstract

Despite the economic importance of caseous lymphadenitis (CLA), a chronic disease caused by *Corynebacterium pseudotuberculosis*, few genes related to the virulence of its etiologic agent have been characterized. The oligopeptide permease (Opp) transporters are located in the plasma membrane and have functions generally related to the uptake of peptides from the extracellular environment. These peptide transporters, in addition to having an important role in cell nutrition, also participate in the regulation of various processes involving intercellular signaling, including the control of the expression of virulence genes in pathogenic bacteria. To study the role of Opp in *C. pseudotuberculosis*, an OppD deficient strain was constructed via simple crossover with a nonreplicative plasmid carrying part of the *oppD* gene sequence. As occurred to the wild-type, the Δ*oppD* strain showed impaired growth when exposed to the toxic glutathione peptide (GSH), indicating two possible scenarios: (i) that this component can be internalized by the bacterium through an Opp-independent pathway or (ii) that there is toxicity while the peptide is extracellular. Additionally, the Δ*oppD* mutant presented a reduced ability to adhere to and infect macrophages compared to the wild-type, although both strains exhibit the same potential to colonize spleens and cause injury and death to infected mice.

## 1. Introduction

*Corynebacterium pseudotuberculosis* is a Gram-positive bacterium and a facultative intracellular parasite that causes caseous lymphadenitis (CLA), a chronic infectious disease affecting small ruminants. The widespread occurrence and economic importance of the disease have stimulated studies on the molecular basis of virulence of this pathogen, on which information is still limited [1]. The genes of* C. pseudotuberculosis* already characterized that are involved in virulence include the* pld* gene, which encodes a powerful exotoxin associated with pathogen dissemination in the host [2, 3], and the operon* fag*ABCD, which is associated with iron absorption [4].

Among the molecular factors associated with the virulence of pathogenic bacteria, the peptide transporters (Opp) have been highlighted, which are multisubunit protein complexes that belong to the family of ABC transporters. This family uses energy generated by the hydrolysis of adenosine triphosphate (ATP) to drive the transport of lipids, peptides, and saccharides across the plasma membrane [5, 6]. These transporters are located in the plasma membrane, and their main function is to capture peptides from the extracellular environment to serve as sources of plasma carbon and nitrogen [5, 6]. In addition to their nutritional role, the peptides can be used by Gram-positive bacteria as signaling molecules in intercellular communication, which allows the bacteria to coordinate the expression of specific genes at a population level. The control of various cellular processes, including sporulation, conjugation, and virulence, has been linked to communication via signal peptides [7–9]. Several studies performed on pathogenic bacteria of the genera* Staphylococcus* sp.,* Streptococcus* sp., and* Mycobacterium* sp. have shown that Opp mutant strains show reduced virulence [7, 10, 11].

The Opp transporters were already characterized in some Gram-positive and Gram-negative bacteria as being composed of five protein subunits generally arranged in operons: OppA, OppB, OppC, OppD, and OppF. Modifications in the gene arrangement, the presence of more than one copy, and the fusion of these subunits have already been observed in the genomes of some bacteria [12]. Generally, the OppA subunit is responsible for the capture of peptides from the extracytoplasmic environment and the transfer of these molecules to transmembrane channel-forming proteins (OppB and OppC). The OppB and OppC subunits are responsible for the formation of the transmembrane channel through which the oligonucleotides are transported to the intracellular environment. The OppD and OppF subunits are located in the cytoplasmic portion of the bacterial membrane and are responsible for ATP hydrolysis, thus generating energy for the peptide internalization process [6, 13].

We studied the role of the Opp transporters in* Corynebacterium pseudotuberculosis* through the generation of a mutant strain with a disruption in the gene* oppD *caused by simple homologous recombination. The knockout of* oppD *causes the inactivation of the entire transport system because this gene encodes the protein responsible for providing energy to the peptide internalization process through the hydrolysis of ATP [14]. After obtaining and confirming the* C. pseudotuberculosis* Δ*oppD* genotype, tests were performed to confirm the loss of function of the transporter. This mutant strain was also assessed for adhesion and viability in macrophages and for virulence in mice to analyze the persistence of infection and the mortality rates.

## 2. Materials and Methods

### 2.1. Bacterial Strains, Plasmids, and Culture Conditions

The bacterial strains used were* Escherichia coli* DH5*α* (Invitrogen) and* Corynebacterium pseudotuberculosis* wild-type strain 1002 [15], and the plasmid used was pCR2.1-TOPO (Invitrogen). The culture media used were Luria-Bertani (LB) (HIMEDIA) for* E. coli* and Brain Heart Infusion (BHI) (HIMEDIA) for* C. pseudotuberculosis*, which were supplemented with 1.5% bacteriological agar for culture on solid medium. Both cultures were grown at 37°C under agitation.* E. coli* was grown for 18 h, and* C. pseudotuberculosis* was grown for 72 h. When necessary, the culture media were supplemented with ampicillin (100 *μ*g/mL) or kanamycin (50 *μ*g/mL).

### 2.2. Construction of the* C. pseudotuberculosis* **Δ** *oppD* Strain

For the construction of* C. pseudotuberculosis* Δ*oppD* by simple homologous recombination [16], a clone of a* C. pseudotuberculosis* genome library generated by D'Afonseca et al. [17] was used, which contained a fragment of the open reading frame (ORF) of the cloned* oppD* gene. Confirmation of the portion of the* oppD* gene cloned into the plasmid was performed by sequencing the fragment and subsequent sequence alignment with the* C. pseudotuberculosis* genome. This search was performed using the Artemis software. After the identification of the* E. coli* clone in the genomic library that contained the* oppD* fragment cloned into pCR2.1-TOPO (Invitrogen), plasmid DNA extraction was performed using the Wizard* Plus* Maxipreps DNA Purification System (Promega). The extracted plasmid was directly transformed into* C. pseudotuberculosis *strain 1002 according to Dorella et al. [18]. The selection of the* oppD* mutant clones was performed in BHI medium supplemented with 50 *μ*g/mL kanamycin. To confirm* oppD* inactivation by the insertion of the suicide vector, polymerase chain reaction (PCR) was performed using primers aligning with* oppD* as well as* m13* and* km*. All other molecular biology techniques were performed according to Sambrook et al. [19].

### 2.3. *In Silico* Analysis of* C. pseudotuberculosis* Opp

Parallel to the construction of* C. pseudotuberculosis* Δ*oppD*, an analysis of the gene content of Opp, its organization, and its similarity to other bacteria was performed using the Artemis software. The operon analyses were performed using SoftBerry (http://linux1.softberry.com/berry.phtml?topic=fgenesb&group=help&subgroup=gfindb). A similarity analysis of the genes contained in the operon was performed using basic local alignment and search tool (BLASTp) (http://blast.ncbi.nlm.nih.gov/Blast.cgi), and only the best hit was used for comparison. An analysis of the protein domains present in the subunits of the transporter was performed through the conserved domain (http://www.ncbi.nlm.nih.gov/Structure/cdd/cdd.shtml).

### 2.4. Growth Curves of the* C. pseudotuberculosis* 1002 Wild-Type and** Δ** *oppD *Strains in the Presence of GSH

Preinocula of* C. pseudotuberculosis* 1002 and Δ*oppD* were prepared in 20 mL of BHI with 0.05% Tween80 (Sigma). After 24 hours of growth at 37°C, aliquots of the cultures were reinoculated (1 : 100) in 120 mL BHI supplemented with Tween80 and incubated at 37°C until the initial exponential growth phase was reached (OD_600 *nm*_ = ~ 0.2). At this time, GSH (Sigma) was added at concentrations of 5 mM and 10 mM to two aliquots of culture, according to Green et al. [20]. The controls received no treatment with GSH. The monitoring of the growth curves was performed by OD_600 nm_ readings at 0, 15, 60, 120, 180, 240, 300, and 360 min after the addition of the substrate, which is toxic to cultures. From the OD_600 nm_ data, growth curves were plotted comparing the wild-type and mutant strains in the presence and absence of the toxic substrate GSH. The OD_600 nm_ data were analyzed with GraphPad Prism v.5.0 (http://www.graphpad.com/scientific-software/prism/).

### 2.5. Viability of* C. pseudotuberculosis *1002 and** Δ** *oppD* in the Murine Macrophages J774

The J774 cell line was grown in Dulbecco's modified eagle's medium (DMEM) (Life Technologies) supplemented with 50 *μ*g/mL gentamicin, 2.5 *μ*g/mL fungizone, and 5% fetal bovine serum (Life Technologies) at 37°C in a CO_2_ incubator. Confluent monolayers were trypsinized at two-day intervals with a saline buffer containing 0.2% (w/v) trypsin and 0.02% (w/v) ethylenediaminetetraacetic acid (EDTA) (Sigma). For the adhesion assays, 500 *μ*L of cell suspension (5 × 10^5^ cells/mL) was distributed into 24-well microplates and incubated in a CO_2_ incubator for 48 hours until the monolayer was 95% confluent. In parallel, the* C. pseudotuberculosis* strains were grown in BHI broth and incubated for 24 hours at 37°C. After three washes of the samples with Dulbecco's phosphate-buffered saline (Life Technologies), the strains were resuspended in DMEM medium to obtain 5 × 10^6^ CFU/mL (multiplicity of infection (MOI) = 10 bacteria* per* macrophage). After the interaction of the bacteria and macrophages for 1, 3, and 6 hours, aliquots of the supernatants were removed, diluted, and plated on BHI agar to count the colony-forming units per milliliter (CFU/mL). The percentage of adherent cells was determined from the sum of the CFUs in the supernatant plus the CFUs associated with monolayers (CFUs associated with monolayers/CFUs in supernatant + CFUs associated with monolayers ×100). The intracellular viability assays were performed with the addition of gentamycin after incubation times of 1, 3, and 6 hours. The percentage of viable intracellular bacteria was determined from the control of associated bacteria (without exposure to gentamicin). The significant difference among the groups was determined using the ANOVA and Tukey's tests of the GraphPad Prism 5.0 (http://www.graphpad.com/scientific-software/prism/). The value of *P* < 0.05 was used as the threshold for significance.

### 2.6. Infection and Persistence Assays of* C. pseudotuberculosis* 1002 and** Δ** *oppD*

The standardization of parameters such as the lethal dose (LD50), volume of the cultures to be inoculated, most appropriate inoculation location, and intervals between immunizations were performed according to Ribeiro et al. [15]. All procedures with animals were carried out according to the regulations of the Ethics Committee for Animal Experimentation of the Federal University of Minas Gerais, Brazil. For experiments evaluating virulence through experimental infection, 6- to 8-week-old BALB/c mice were divided into three groups of 15 animals each. One group was used as a negative control and inoculated with 0.9% saline solution. The second group was infected with the* C. pseudotuberculosis* 1002 strain, and the third was infected with* C. pseudotuberculosis* Δ*oppD*. All animals were infected intraperitoneally with a final volume of 100 mL for each dose. The inoculations with both strains contained 10^6^ CFU/mL. The animals were evaluated daily for the formation of abscesses at the site of injection, the degree of prostration, and the number of deaths. The survival rates of the animals were calculated and represented in GraphPad Prism v.5.0 (http://www.graphpad.com/scientific-software/prism/) using the Kaplan-Meier survival function. For infection persistence assays, we proceeded in the same manner as described above, except that the inoculation dose was slightly lower than the previous one (5 × 10^5^ CFU/mL), to prevent the animals from dying before the completion of the experiment. During the period of 1–5 days after infection, three animals from each group were sacrificed, and their spleens were removed and macerated in 2 mL of 0.9% saline solution. From this macerate, 10^−1^ and 10^−2^ dilutions were plated on BHI plates for the mice infected with the wild-type strain and on BHI supplemented with 50 mg/mL of kanamycin (Sigma) for the mice infected with the mutant strain. The results of the* C. pseudotuberculosis* CFU count in the spleen were calculated and represented in GraphPad Prism v.5.0 (http://www.graphpad.com/scientific-software/prism/) using the two-way ANOVA test.

## 3. Results and Discussion

### 3.1. Generation and Confirmation of* C. pseudotuberculosis* **Δ** *oppD*

With the mapping of a genomic library constructed by D'Afonseca et al. [17], it was possible to identify and sequence in one of the clones a 1,200 bp fragment from the ORF of the* oppD* gene ligated into the plasmid pCR2.1-TOPO. The nucleotide sequence of the chromosomal DNA fragment of* C. pseudotuberculosis* ligated into the plasmid pCR2.1-TOPO in one of the clones of the genomic library of* C. pseudotuberculosis* had 100% similarity to 1,000 bp of the* oppD*(see Figure S1 in Supplementary Material available online at http://dx.doi.org/10.1155/2014/489782). After transformation into* C. pseudotuberculosis*, the mutant strain showed resistance to kanamycin, and their DNA was subjected to PCR with various primer combinations (Table S1).

### 3.2. *In Silico* Characterization of the* opp* Operon of* C. pseudotuberculosis*

**Figure 1:**
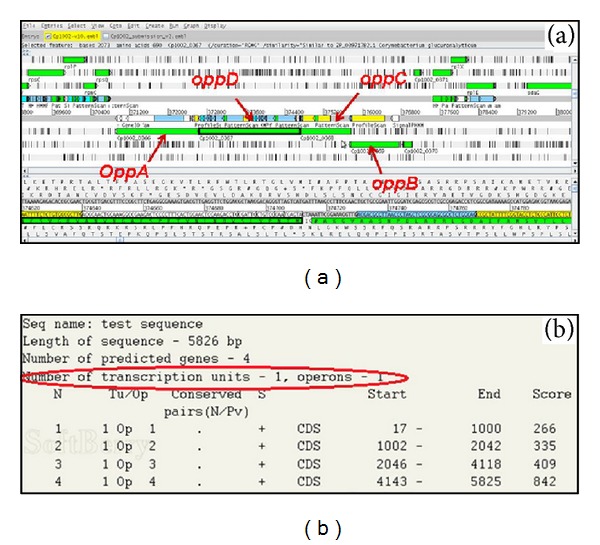
*In silico* analysis of the operon* oppBCDA* in the genome of* C. pseudotuberculosis*. (a) Organization of operon (visualized by software Artemis). Arrows indicate the arrangement of genes in the genome. (b) Prediction of* oppBCDA* operon by software FGENESB. The red circle indicates that the genes are transcribed into a single transcriptional unit.

Through* in silico* analysis, we found that the Opp transporter is part of the core of* C. pseudotuberculosis*, and it likely has an essential role in the biology of the organism. A sequence analysis of the genes encoding the protein subunits of the Opp also showed that they are part of an operon and are transcribed in a single transcriptional unit (Figure 1). In an* in silico* protein analysis, a protein domain of OppA was found to be similar to an extracellular peptide-binding domain, which may be related to the capture of peptides from the extracytoplasmic environment and the transfer of these peptides to the transmembrane channel-forming proteins. Moreover, the amino acid sequences of OppB and OppC showed conserved domains related to the formation of transport channels across the plasma membrane. With respect to OppD, two protein domains related to ATP hydrolysis (Figure S2) were found, which may indicate a fusion between the* oppD* and* oppF* genes and explain the absence of the* oppF* gene in the operon in* C. pseudotuberculosis*. This same configuration was found in* Mycobacterium tuberculosis*, demonstrating that this fusion may be a characteristic of actinobacteria [6].

### 3.3. Phenotypic Test to Evaluate the Peptide Internalization of* C. pseudotuberculosis* **Δ** *oppD*

**Figure 2:**
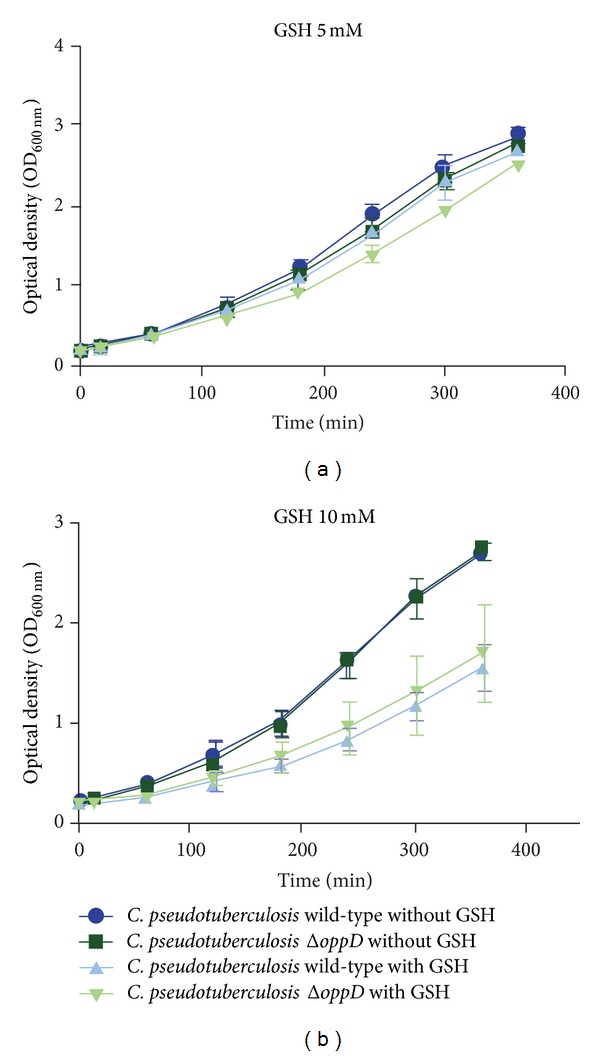
Growth of wild-type and Δ*oppD C. pseudotuberculosis* in the presence or absence of GSH. (a) Phenotypic test with 5 mM GSH and (b) phenotypic test with 10 mM GSH.

One method by which to phenotypically characterize mutant strains defective in the transport of molecules across the plasma membrane is to use substrate analogs that are toxic to the bacterium when internalized by these transporters. This methodology is effective because it is expected that the absence of the transporter in the mutant strain makes this strain resistant to the effects of the toxic substrate given that the substrate will not be internalized by the bacterium. Conversely, in the wild-type strain, the presence of the functional transporter will enable the toxic substrate to be transported into the cell, thus causing damage to the bacterium. The use of GSH as a toxic substrate proved to be an effective tool in the characterization of mutant strains for the Opp peptide transporter in* Mycobacterium bovis* (phylogenetically close to* C. pseudotuberculosis*) [20]. Thus, we used the same molecule for the phenotypic characterization of* C. pseudotuberculosis* Δ*oppD*, and the results are shown in Figure 2. Unlike the study by Green et al. [20] on* Mycobacterium bovis* Δ*oppD*, in which the wild-type strain was sensitive to concentrations of 5 mM of GSH,* C. pseudotuberculosis* was resistant to the toxic effects of GSH at this concentration because the wild-type and mutant strains showed the same growth profile in the presence and absence of the toxic substrate (Figure 2(a)). With the aim of finding a GSH concentration in which* C. pseudotuberculosis* was sensitive to the toxic effects of this substrate, various concentrations of GSH were tested (data not shown), and the concentration chosen was 10 mM given that concentrations above this value inhibited the growth of both strains.  Figure 2(b) shows that although GSH is toxic to* C. pseudotuberculosis* at the concentration of 10 mM, the use of this substrate did not allow us to phenotypically distinguish the wild-type and mutant strains because the growth of both was affected in the same way by the presence of GSH at that concentration. Two hypotheses were raised from these results: (i) GSH does not need to be transported to the cytoplasm of* C. pseudotuberculosis* to be toxic to the bacterium or (ii) GSH is being internalized by the bacterium through a pathway different from Opp.

### 3.4. Infection of Macrophages by* C. pseudotuberculosis* **Δ** *oppD*

**Figure 3:**
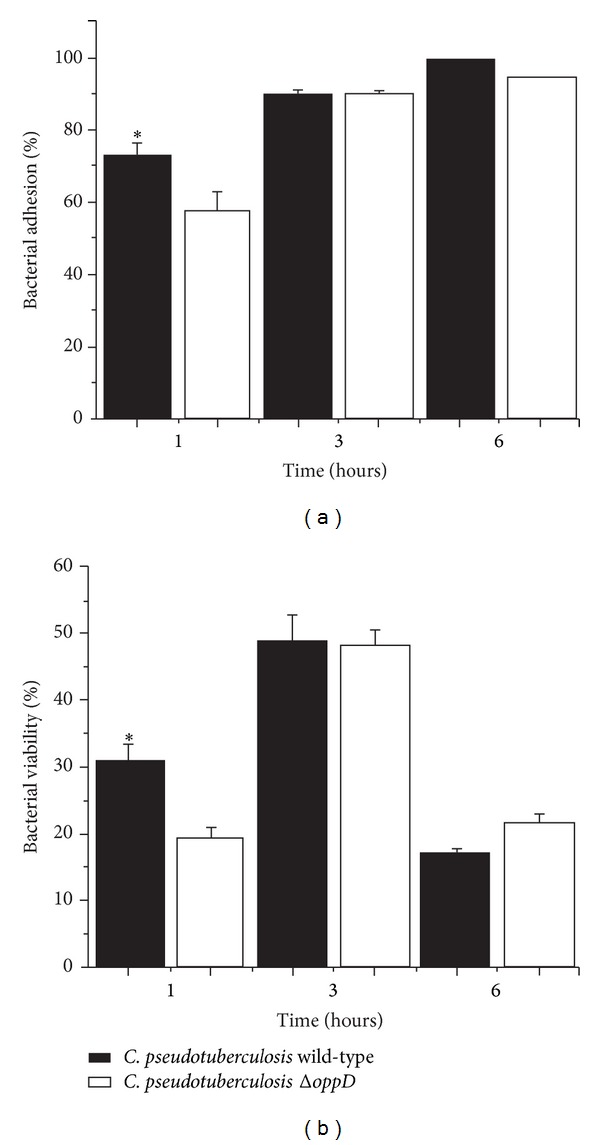
Adhesion and internalization of wild-type* C. pseudotuberculosis* and Δ*oppD* mutant into J774 macrophages. (a) Bacterial adherence of wild-type and mutant strains was evaluated 1, 3, and 6 hours after infection. (b) Viability of wild-type and mutant strains inside macrophages was evaluated 3 and 6 hours after infection. Asterisks indicate significant differences between groups tested (*P* < 0.05).

In the course of the infection by* C. pseudotuberculosis*, the bacterium needs to be internalized to survive in this environment as an intracellular parasite. The tests performed to evaluate whether these characteristics are still present in* C. pseudotuberculosis* Δ*oppD* showed a significant reduction in adhesion only during the first hour, and at 3 and 6 hours, both strains showed the same ability to adhere to and infect macrophages (Figure 3(a)). These results suggest that* C. pseudotuberculosis* Δ*oppD* exhibits a delay in the ability to adhere to the cell membranes of macrophages because only during the initial phases of the experiment the percentage of adherent mutant bacteria was lower than that of the wild-type bacteria. The lower adhesion potential of the mutant strain also led to a decline in internalization (Figure 3(b)) given that, to infect the phagocytic cell, the bacteria must first establish contact with the macrophage membrane. Studies conducted on different species of the genus* Streptococcus* demonstrated that Opp mutant strains showed a reduced ability to adhere to human cells [21, 22]. This reduction in* Streptococcus pyogenes* was caused by a decrease in the expression of* fbsA*, which encodes a fibrinogen-binding adhesin [9]. Thus, Opp may be associated with the transcriptional control of genes related to bacterial adhesion.

### 3.5. Virulence of* C. pseudotuberculosis* **Δ** *oppD* in BALB/c Mice

**Figure 4:**
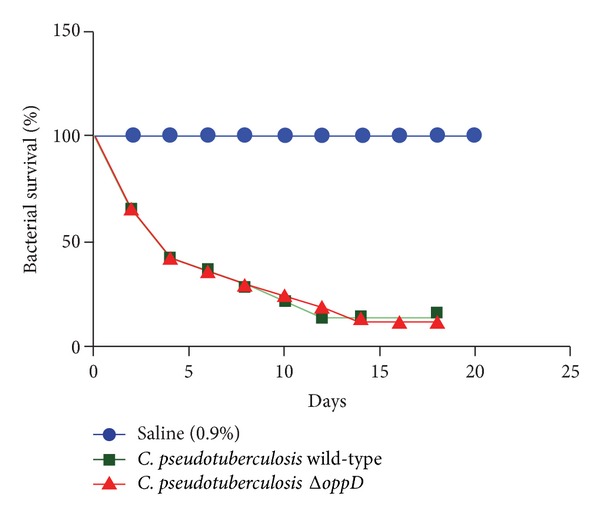
Survival curve of BALB/c mice infected with* C. pseudotuberculosis* Δ*oppD.*

**Figure 5:**
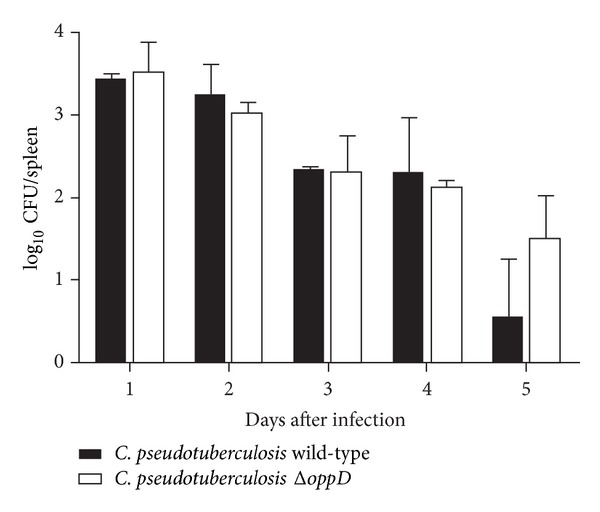
Colony-forming units in the spleen of BALB/c mice infected with wild-type* C. pseudotuberculosis* and Δ*oppD* mutant for the first 5 days of infection.

To assess whether the inactivation of the* oppD* gene in* C. pseudotuberculosis* resulted in a decrease in the virulence profile of the mutant strain, infection assays using BALB/c mice were performed.  Figure 4 shows that there was no significant difference between the survival curves of animals infected with* C. pseudotuberculosis* 1002 and those infected with* C. pseudotuberculosis* Δ*oppD*, corroborating the general physical examination of the mice that also showed no difference between the infected groups. This same result was observed by Flores-Valdez et al. [23] in a study performed with* Mycobacterium tuberculosis* in which the virulence of an Opp mutant strain was evaluated through infection of BALB/c mice via aerosol. That study revealed that the mutant strain showed no significant difference in the ability to cause death in mice compared to the wild-type strain. However, when evaluating the colonization potential of the mutant strain in organs such as the spleen and lung during the acute, chronic, and persistent phases of infection, a lower bacterial load was observed during the chronic phase of infection in mice inoculated with this mutant strain. Unlike infections caused by* M. tuberculosis* in mice, in infections by* C. pseudotuberculosis*, it is only possible to recover live bacteria from the spleen of infected mice six days after inoculation. Thus, the assays designed to evaluate the ability to colonize the spleen of mice infected with* C. pseudotuberculosis* cover only the acute phase. To assess if there was a reduction in the potential for spleen colonization during the infection of mice with* C. pseudotuberculosis* Δ*oppD*, a comparison was performed between the number of CFUs of the wild-type and mutant strains recovered from the spleens of the mice during the acute phase of infection.  Figure 5 demonstrates that the bacterial load found in the spleens of mice infected with the mutant strain was not significantly different from that found in animals infected with the wild-type strain. Borezee et al. [24], in a study performed with* Listeria monocytogenes* Δ*oppA*, observed a reduced growth capacity of this bacterium in murine macrophages compared to the wild-type strain. Despite this reduction, the mutant strain showed the same ability as the wild-type strain to kill the infected mice. Unlike the results of studies with other pathogenic bacteria such as* S. aureus*,* M. tuberculosis*, and* S. pyogenes*, in which the Opp mutant strains showed reductions in virulence and pathogenicity in assays of mice infection [10, 11, 24], in* C. pseudotuberculosis*, the presence of a functional Opp transporter appears not to be required for the virulence of this bacterium in this infection model.

## 4. Conclusions

This study is the first report on the role of the Opp transporter in bacteria of the genus* Corynebacterium* spp. We characterized the* Opp* operon and evaluated its importance in the virulence and pathogenicity of* C. pseudotuberculosis*. The phenotypic tests using GSH showed that Opp is not necessary for the internalization of peptides, which can use an alternative pathway. Although the* in vitro* assays of bacterial adhesion and viability in murine macrophages demonstrate that* C. pseudotuberculosis* Δ*oppD* exhibits a delay in the adhesion to the cell membrane compared to the wild-type strain, this delay did not affect the virulence of the Δ*oppD* strain in mice.

## Supplementary Material

For the construction of C. pseudotuberculosis *δoppD* by simple homologous recombination, a clone of a C. pseudotuberculosis genome library generated by D'Afonseca et al. [17] was used, which contained a fragment of the Open Reading Frame (ORF) of the cloned *oppD* gene. Confirmation of the portion of the *oppD* gene cloned into the plasmid was performed by sequencing the fragment and subsequent sequence alignment with the *C. pseudotuberculosis* genome (Figure S1). This search was performed using the Artemis software. After the identification of the *E. coli* clone in the genomic library that contained the *oppD* fragment cloned into pCR®2.1-TOPO®, plasmid DNA extraction was performed using the Wizard®Plus Maxipreps DNA Purification System. The extracted plasmid was directly transformed into C. pseudotuberculosis strain 1002 according to Dorella et al. [18]. The selection of the *oppD* mutant clones was performed in BHI medium supplemented with 50 µg/mL kanamycin. To confirm *oppD* inactivation by the insertion of the suicide vector, Polymerase Chain Reaction (PCR) was performed using primers aligning to *oppD* as well as *m13* and *km* (Table S1) All other molecular biology techniques were performed according to Sambrook [19].
