# Supplementary figures and images for: Characterization of the Opp Peptide Transporter of Corynebacterium pseudotuberculosis and Its Role in Virulence and Pathogenicity
Source: Biomed Res Int. 2014 May 8;2014:489782. doi: 10.1155/2014/489782 (PMC4034477; doi:10.1155/2014/489782)

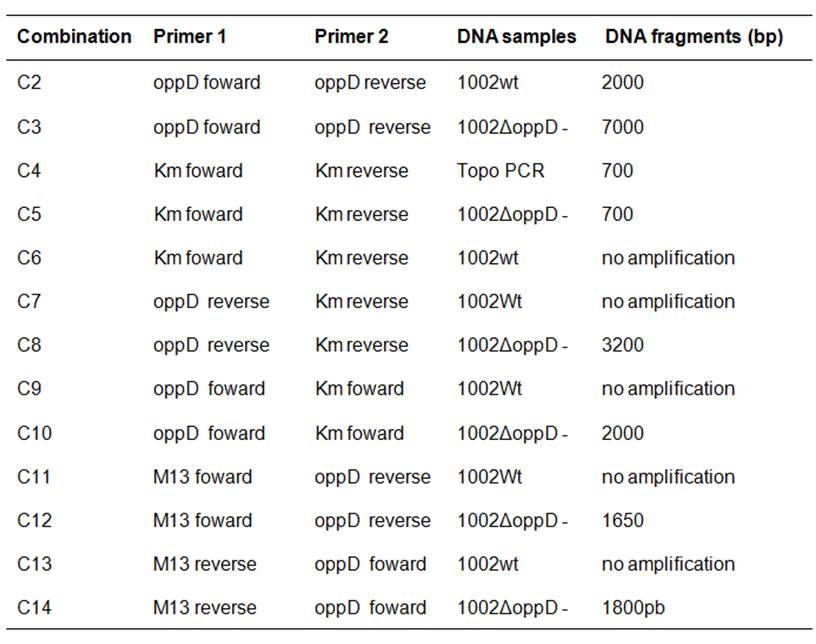

Supplement: Supplementary file 1 — For the construction of C. pseudotuberculosis δoppD by simple homologous recombination, a clone of a C. pseudotuberculosis genome library generated by D'Afonseca et al. [17] was used, which contained a fragment of the Open Reading Frame (ORF) of the cloned oppD gene. Confirmation of the portion of the oppD gene cloned into the plasmid was performed by sequencing the fragment and subsequent sequence alignment with the C. pseudotuberculosis genome (Figure S1). This search was performed using the Artemis software. After the identification of the E. coli clone in the genomic library that contained the oppD fragment cloned into pCR®2.1-TOPO®, plasmid DNA extraction was performed using the Wizard®Plus Maxipreps DNA Purification System. The extracted plasmid was directly transformed into C. pseudotuberculosis strain 1002 according to Dorella et al. [18]. The selection of the oppD mutant clones was performed in BHI medium supplemented with 50 µg/mL kanamycin. To confirm oppD inactivation by the insertion of the suicide vector, Polymerase Chain Reaction (PCR) was performed using primers aligning to oppD as well as m13 and km (Table S1) All other molecular biology techniques were performed according to Sambrook [19]. [file 489782.f1.zip › Table_S1_2738_904589.jpg]

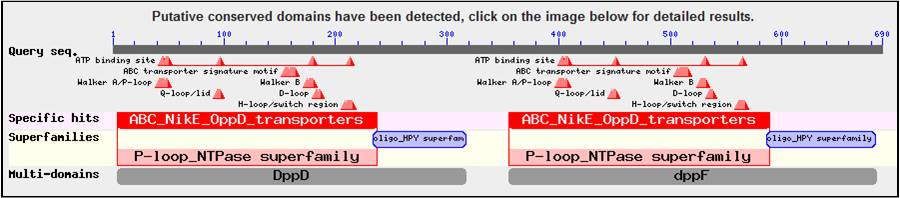

Supplement: Supplementary file 1 — For the construction of C. pseudotuberculosis δoppD by simple homologous recombination, a clone of a C. pseudotuberculosis genome library generated by D'Afonseca et al. [17] was used, which contained a fragment of the Open Reading Frame (ORF) of the cloned oppD gene. Confirmation of the portion of the oppD gene cloned into the plasmid was performed by sequencing the fragment and subsequent sequence alignment with the C. pseudotuberculosis genome (Figure S1). This search was performed using the Artemis software. After the identification of the E. coli clone in the genomic library that contained the oppD fragment cloned into pCR®2.1-TOPO®, plasmid DNA extraction was performed using the Wizard®Plus Maxipreps DNA Purification System. The extracted plasmid was directly transformed into C. pseudotuberculosis strain 1002 according to Dorella et al. [18]. The selection of the oppD mutant clones was performed in BHI medium supplemented with 50 µg/mL kanamycin. To confirm oppD inactivation by the insertion of the suicide vector, Polymerase Chain Reaction (PCR) was performed using primers aligning to oppD as well as m13 and km (Table S1) All other molecular biology techniques were performed according to Sambrook [19]. [file 489782.f1.zip › Figure_S2_2738_904588.jpg]
