# Supplementary material for: Characterization of the Opp Peptide Transporter of Corynebacterium pseudotuberculosis and Its Role in Virulence and Pathogenicity
Source: Biomed Res Int. 2014 May 8;2014:489782. doi: 10.1155/2014/489782 (PMC4034477; doi:10.1155/2014/489782)
Supplement: Supplementary file 1 — For the construction of C. pseudotuberculosis δoppD by simple homologous recombination, a clone of a C. pseudotuberculosis genome library generated by D'Afonseca et al. [17] was used, which contained a fragment of the Open Reading Frame (ORF) of the cloned oppD gene. Confirmation of the portion of the oppD gene cloned into the plasmid was performed by sequencing the fragment and subsequent sequence alignment with the C. pseudotuberculosis genome (Figure S1). This search was performed using the Artemis software. After the identification of the E. coli clone in the genomic library that contained the oppD fragment cloned into pCR®2.1-TOPO®, plasmid DNA extraction was performed using the Wizard®Plus Maxipreps DNA Purification System. The extracted plasmid was directly transformed into C. pseudotuberculosis strain 1002 according to Dorella et al. [18]. The selection of the oppD mutant clones was performed in BHI medium supplemented with 50 µg/mL kanamycin. To confirm oppD inactivation by the insertion of the suicide vector, Polymerase Chain Reaction (PCR) was performed using primers aligning to oppD as well as m13 and km (Table S1) All other molecular biology techniques were performed according to Sambrook [19]. [file 489782.f1.zip › Figure S1.doc]

**Figure S1A.**

**GGGAGGGTNACGACGGCAGTGATCCAATAGGTTANACTATAGGGCGAATT**

**GAATCAGCGGCCGTGAATCGCCCTTACCGCAGACAACGTCATGGTTATGT**

**ATGCAGGACGCCCTGTAGAATACGCCGGCGTCATGAGCTCTTCAGTGAGC**

**CCAAAATGCCTTATACCGTCGGCTTGCTTGGTTCTATTCCCTCCGTGCGC**

**AAACGTGAGAAAGTCTCCCTCACCACTATTGAGGGCAGCCCTCCTATCGT**

**GGTTAATCTTCCCGACGAATGCTCCTTCGCTCCCCGCTGCCCTATAGCTA**

**CAGCCGAATGCCTTAAAACTGAGCCCGGGCTCTTATCCATCAATGATAAG**

**CACCAGGCGGCATGCATCCGTTCTACTGAGATCAGCCATGGAGAACTTAA**

**CGGCAGCTCCGATTTTCCCCACGCCGCCCGTTCACGAGGGTGCTCTCATG**

**CGGATTCCTCGGGAAAAGCGCTCCTGCGTTCTCAGCGTCTCTGACCTGAC**

**TAAAACATTTCCTTTGGTCAAAGGCAGCCCTGCTTAAACGAACTTGTGGG**

**AGAAGTGCGGGCCGTTAACGGTTTAACTTTTGATATCAAAGAAGGCGAGT**

**GCTTTGCTATCGTCGGCGAATCCGGATGCGGGAAAACCACCACCTTGCTG**

**GAGATCATGGATCTAGCCCCGCAAGCGCCGATCAAATCCAATTAGGTGAG**

**AAAGACCGTCGCAGGCATCTCCAAGCGTCAACGGACGTCTTCTAACGAAN**

**ACGACCATTCAAAATTGTACCAAGAACCCATGGNGGGGCTCTTGAATCAC**

**NGGGGGCCATCTCAAACTGGGTCGGCACAGAAAGAAATTACCTCCCCGGG**

**GAAAGGACCCTCCNTGGGAAAATNTCCCTTGGAGGGGCCTAGGGGGAANA**

**CGGNAAAACAAAAATGGAACGCGGAGATCACCGGNAAAAACTCAATCCAC**

**GANAAACAGACCGGGTAAAACCCCCGTGGCACAATTACACAATCACNAAG**

**TAACACACGNGAGCACACN**

**CAGTATAGCAGCTCAGAATACCCTCAGGATTAGGACTAGTCCTGCAGGTT**

**TAAACGAATTCGTTACCTTACATCACGGCGGCACGGTCTGAGATATGCCG**

**AATAACGGAGAGATCGTGAGCCACAAAAAGATAGCTGATACCCAGGCGCG**

**CTTTGAGCTCATCAAGGAGATTCAGCATTCCGGCCTGGACAGAGACGTCC**

**AGGGCAGACACAGGCTCATCAAGAACAATCAACTTGGGATTGGCCGCAAG**

**CGCACGGGCAAGAGATATACGCTGGCGTTGTCCACCGGAAAAATGGCCAG**

**GGAACCTAATCAATATGAATATGAGCAGGGTTTAGCCCGACTAACTGGAT**

**GAGCTCGCGGATACGCTCATTTTTATCGCCATCCCAGCCCAAGGAATCCA**

**GAGGCTCCCGGAGAATCTCTGCGACAGTGAGGCGCGGATCAAGAGCCCCC**

**ATGGGGTCTTGGAAAACAATTTGAATGTCTTTTCTTAGAAGACGCCGTTG**

**ACGCTTGGAGATGCCTGCGACGTCTTTCTCACCTAATTGGATTTGAGTCG**

**GCGCTTGCGGGGCTAGATCCATGATCTCCAGCAAGGTGGTGGTTTTNCCC**

**GCATCCGGATTCGCCGACGATAGCAAAGCACTCGCCTTCTTTAGATATCA**

**AAAGTTAAACCGTTAACGGCCCGCACTTCTCCCACAGATCGTTAAGCAGG**

**GCGCCTTTGACCAAAGGANAATGTTAAGTCAGGTCAAGAGACGCTGAGAA**

**CGCANGAGACGCTTTTCCCCGAGGGCAATCCCGCATGAGAGCACCACTCG**

**TTGGAACAGGGCGGCCGTGGGNGCAAAATCGGGAACACGNCCCGAGTTAA**

**AGNTTACCACAAGGGGTGGANCTCACGTAAAAACGGAATTGCCATTGCAC**

**GGNCCTGGGTGGCTTAATAACATGGGACAGGATTACAAAGCACCGGGANC**

**CAAAGTAAGGGCACANCCGCNNCGTAAGAAAAANAGGAACAAGCGGAAAA**

**CNACAAGGAACATGAACGGGAAATACACNA**

**Figure S1B.**

```
BLASTN

Reference: Altschul, Stephen F., Thomas L. Madden, Alejandro A. Schaffer,
Jinghui Zhang, Zheng Zhang, Webb Miller, and David J. Lipman (1997),
"Gapped BLAST and PSI-BLAST: a new generation of protein database search
programs",  Nucleic Acids Res. 25:3389-3402.

Query= Contig  oppD (CAP3)
Database: Cp 1 sequences; 2,496,273 total letters
Score = 2133 bits (1076), Expect = 0.0 /Identities = 1083/1084 (99%),Strand = Plus / Minus


Query: 45     taccgcagacaacgtcatggttatgtatgcaggacgccctgtagaatacgccggcgttca 104
              ||||||||||||||||||||||||||||||||||||||||||||||||||||||||||||
Sbjct: 477518 taccgcagacaacgtcatggttatgtatgcaggacgccctgtagaatacgccggcgttca 477459

Query: 105    tgagctcttcagtgagcccaaaatgccttataccgtcggcttgcttggttctattccctc 164
              ||||||||||||||||||||||||||||||||||||||||||||||||||||||||||||
Sbjct: 477458 tgagctcttcagtgagcccaaaatgccttataccgtcggcttgcttggttctattccctc 477399

Query: 165    cgtgcgcaaacgtgagaaagtctccctcaccactattgagggcagccctcctatcgtggt 224
              ||||||||||||||||||||||||||||||||||||||||||||||||||||||||||||
Sbjct: 477398 cgtgcgcaaacgtgagaaagtctccctcaccactattgagggcagccctcctatcgtggt 477339

Query: 225    taatcttcccgacgaatgctccttcgctccccgctgccctatagctacagccgaatgcct 284
              ||||||||||||||||||||||||||||||||||||||||||||||||||||||||||||
Sbjct: 477338 taatcttcccgacgaatgctccttcgctccccgctgccctatagctacagccgaatgcct 477279

Query: 285    taaaactgagcccgggctcttatccatcaatgataagcaccaggcggcatgcatccgttc 344
              ||||||||||||||||||||||||||||||||||||||||||||||||||||||||||||
Sbjct: 477278 taaaactgagcccgggctcttatccatcaatgataagcaccaggcggcatgcatccgttc 477219

Query: 345    tactgagatcagccatggagaacttaacggcgctccgattttccccacgccgcccgttca 404
              ||||||||||||||||||||||||||||||||||||||||||||||||||||||||||||
Sbjct: 477218 tactgagatcagccatggagaacttaacggcgctccgattttccccacgccgcccgttca 477159

Query: 405    cgagggtgctctcatgcggattcctcgggaaaagcgctcctgcgttctcagcgtctctga 464
              ||||||||||||||||||||||||||||||||||||||||||||||||||||||||||||
Sbjct: 477158 cgagggtgctctcatgcggattcctcgggaaaagcgctcctgcgttctcagcgtctctga 477099

Query: 465    cctgactaaaacatttcctttggtcaaaggcgccctgcttaaacgaactgtgggagaagt 524
              ||||||||||||||||||||||||||||||||||||||||||||||||||||||||||||
Sbjct: 477098 cctgactaaaacatttcctttggtcaaaggcgccctgcttaaacgaactgtgggagaagt 477039

Query: 525    gcgggccgttaacggtttaacttttgatatcaaagaaggcgagtgctttgctatcgtcgg 584
              ||||||||||||||||||||||||||||||||||||||||||||||||||||||||||||
Sbjct: 477038 gcgggccgttaacggtttaacttttgatatcaaagaaggcgagtgctttgctatcgtcgg 476979

Query: 585    cgaatccggatgcgggaaaaccaccaccttgctggagatcatggatctagccccgcaagc 644
              ||||||||||||||||||||||||||||||||||||||||||||||||||||||||||||
Sbjct: 476978 cgaatccggatgcgggaaaaccaccaccttgctggagatcatggatctagccccgcaagc 476919

Query: 645    gccgactcaaatccaattaggtgagaaagacgtcgcaggcatctccaagcgtcaacggcg 704
              ||||||||||||||||||||||||||||||||||||||||||||||||||||||||||||
Sbjct: 476918 gccgactcaaatccaattaggtgagaaagacgtcgcaggcatctccaagcgtcaacggcg 476859

Query: 705    tcttctaagaaaagacattcaaattgttttccaagaccccatgggggctcttgatccgcg 764
              ||||||||||||||||||||||||||||||||||||||||||||||||||||||||||||
Sbjct: 476858 tcttctaagaaaagacattcaaattgttttccaagaccccatgggggctcttgatccgcg

Query: 765    cctcactgtcgcagagattctccgggagcctctggattccttgggctgggatggcgataa 824
              ||||||||||||||||||||||||||||||||||||||||||||||||||||||||||||
Sbjct: 476798 cctcactgtcgcagagattctccgggagcctctggattccttgggctgggatggcgataa 476739

Query: 825    aaatgagcgtatccgcgagctcatccagttagtcgggctaaaccctgctcatattcatat 884
              ||||||||||||||||||||||||||||||||||||||||||||||||||||||||||||
Sbjct: 476738 aaatgagcgtatccgcgagctcatccagttagtcgggctaaaccctgctcatattcatat 476679

Query: 885    tgataggttccctggccatttttccggtggacaacgccagcgtatatctcttgcccgtgc 944
              ||||||||||||||||||||||||||||||||||||||||||||||||||||||||||||
Sbjct: 476678 tgataggttccctggccatttttccggtggacaacgccagcgtatatctcttgcccgtgc 476619

Query: 945    gcttgcggccaatcccaagttgattgttcttgatgagcctgtgtctgccctggacgtctc 1004
              ||||||||||||||||||||||||||||||||||||||||||||||||||||||||||||
Sbjct: 476618 gcttgcggccaatcccaagttgattgttcttgatgagcctgtgtctgccctggacgtctc 476559

Query: 1005   tgtccaggccggaatgctgaatctccttgatgagctcaaagcgcgcctgggtatcagcta 1064
              ||||||||||||||||||||||||||||||||||||||||||||||||||||||||||||
Sbjct: 476558 tgtccaggccggaatgctgaatctccttgatgagctcaaagcgcgcctgggtatcagcta 476499

Query: 1065   tctttttgtggctcacgatctctccgttattcggcatattctcagaccgtgccgccgtga 1124
              |||||||||||||||||||||||||||||||||||||| |||||||||||||||||||||
Sbjct: 476498 tctttttgtggctcacgatctctccgttattcggcata-tctcagaccgtgccgccgtga 476440

Query: 1125   tgta 1128
              ||||
Sbjct: 476439 tgta 476436
```
